# Reconsiderando o Escore CHA_2_DS_2_-VASc para Estratificação de Risco de Não-Refluxo após Intervenção Coronária Percutânea

**DOI:** 10.36660/abc.20260022

**Published:** 2026-02-26

**Authors:** Irfan Sahin, Muhammed Furkan Deniz

**Affiliations:** 1 Bagcilar Training and Research Hospital Istambul Turquia Bagcilar Training and Research Hospital, Istambul – Turquia

**Keywords:** Intervenção Coronária Percutânea, Complicações Pós-Operatórias, Fenômeno de não Refluxo

O fenômeno de não-refluxo continua sendo uma das complicações mais complexas e clinicamente desafiadoras da intervenção coronária percutânea (ICP).^1 , 2^ Embora técnicas avançadas de imagem, novas tecnologias de stents e terapias farmacológicas contemporâneas sejam amplamente utilizadas hoje em dia, ainda não existe um sistema de pontuação universalmente aceito capaz de prever de forma confiável o não-refluxo antes ou durante o procedimento. Essa lacuna continua sendo uma importante necessidade clínica não atendida.^1 - 4^ Nesse contexto, a revisão sistemática e meta-análise apresentada por Ozbay et al. contribui significativamente para a literatura existente ao abordar a associação entre o escore CHA_2_DS_2_-VASc e a ocorrência de não-refluxo após ICP.^3^ Ao sintetizar dados de dez estudos observacionais envolvendo mais de 6.500 pacientes, os autores demonstraram uma associação significativa e consistente entre escores CHA_2_DS_2_-VASc mais altos e uma maior probabilidade de não-refluxo. Esses achados sugerem que observações relatadas anteriormente em estudos de centro único com tamanhos de amostra relativamente pequenos são confirmadas em uma população maior dentro de uma abordagem metodológica mais robusta.^4 - 11^

O papel potencial do escore CHA_2_DS_2_-VASc na previsão de não-refluxo é apoiado não apenas por associações estatísticas, mas também por forte concordância fisiopatológica. O escore incorpora componentes como idade avançada, hipertensão, diabetes mellitus, insuficiência cardíaca congestiva, doença vascular, acidente vascular cerebral prévio e sexo feminino. Todas essas características clínicas estão intimamente ligadas à disfunção endotelial, inflamação crônica, aumento da tendência trombótica e doença microvascular.^4 , 12^ Considerando que a embolização distal, a obstrução microvascular, a vasoconstrição e a lesão de reperfusão são mecanismos-chave envolvidos na patogênese do fenômeno de não-refluxo, o escore CHA_2_DS_2_-VASc pode servir como um substituto clínico abrangente que reflete esses processos subjacentes.^2^ Portanto, o uso desse escore no contexto do não-refluxo pós-ICP deve ser visto não como uma extensão incidental, mas como um reposicionamento biologicamente significativo.

Um dos pontos fortes notáveis da meta-análise é a consistência geral dos resultados em diferentes subgrupos clínicos. Apesar de um alto grau de heterogeneidade, a manutenção da direção do efeito nas análises de sensibilidade e nas múltiplas avaliações de subgrupos sugere que a associação observada reflete um padrão sistemático, em vez de um achado fortuito.^3^ Análises de subgrupos limitadas a pacientes com infarto agudo do miocárdio com supradesnivelamento do segmento ST (IAMCSST) também demonstraram uma associação persistente e significativa entre o escore CHA_2_DS_2_-VASc e o não-refluxo.^5 - 7^ Além disso, a redução da heterogeneidade e a manutenção da significância estatística após a exclusão de estudos que relataram tamanhos de efeito extremos reforçam ainda mais a robustez das descobertas.

Embora os dados sobre pacientes com infarto do miocárdio sem supradesnivelamento do segmento ST (IAMSSST) fossem mais limitados, as evidências disponíveis mostraram uma tendência consistente com os resultados gerais da meta-análise. Estudos que demonstram uma associação entre o escore CHA_2_DS_2_-VASc e o fenômeno de não-refluxo em populações com IAMSSST requerem interpretação cautelosa devido ao tamanho reduzido das amostras e à natureza observacional dos dados. Essas descobertas apoiam a interpretação de que o escore CHA_2_DS_2_-VASc pode servir como um marcador de risco em diferentes apresentações clínicas dentro do espectro da síndrome coronariana aguda.

No entanto, uma interpretação cuidadosa dessas descobertas é necessária. A natureza observacional de todos os estudos incluídos torna a análise suscetível a fatores de confusão residuais e não mensurados. Variáveis como técnicas de procedimento, experiência do operador, carga trombótica, morfologia da lesão, terapias farmacológicas adjuvantes e estratégias destinadas a reduzir a microembolização não foram relatadas de forma uniforme entre os estudos.^3^ Além disso, variações na definição de não-refluxo e diferenças nas abordagens de modelagem estatística representam importantes fontes de heterogeneidade.^3 , 11 , 13^ A presença de viés de publicação e efeitos de pequenos estudos também não pode ser totalmente excluída.

À luz dessas limitações, fica claro que o escore CHA_2_DS_2_-VASc não deve ser considerado um determinante independente ou uma ferramenta de tomada de decisão para o não-refluxo. Em vez disso, deve ser considerado um indicador clínico prático que reflete a carga cumulativa de risco vascular e a suscetibilidade à lesão microvascular. Nesse contexto, o escore CHA_2_DS_2_-VASc surge como uma ferramenta adequada para o enriquecimento de risco, em vez de um teste diagnóstico.^3 , 11^

Do ponto de vista clínico, em pacientes com escores CHA_2_DS_2_-VASc mais elevados, o escore pode servir como um sinal de alerta precoce, incentivando um planejamento de procedimento mais meticuloso, a implementação de estratégias para minimizar a embolização distal e um monitoramento pós-procedimento mais rigoroso.^3 , 9^

Estudos futuros devem investigar se a integração de escores clínicos, como o CHA_2_DS_2_-VASc, com achados angiográficos, variáveis do procedimento e biomarcadores inflamatórios ou trombóticos pode melhorar ainda mais a previsão de não-refluxo.^3^ Mais importante, são necessários estudos prospectivos randomizados bem delineados para determinar se esses modelos de risco integrados se traduzem não apenas em maior precisão preditiva, mas também em melhores resultados clínicos.

Em conclusão, a meta-análise de Ozbay et al. demonstra uma associação significativa e consistente entre o escore CHA_2_DS_2_-VASc e o desenvolvimento de não-refluxo após ICP.^3^ Essa descoberta reforça ainda mais a estreita relação entre o risco vascular sistêmico e a lesão microvascular e sugere que o escore CHA_2_DS_2_-VASc pode ajudar a identificar pacientes com maior risco de não-refluxo após ICP. Dada a etiologia multifatorial do não-refluxo e sua associação com desfechos clínicos graves, o escore CHA_2_DS_2_-VASc pode representar uma ferramenta útil para identificar pacientes de alto risco.
