# Supplementary material for: Reconsiderando o Escore CHA2DS2-VASc para Estratificação de Risco de Não-Refluxo após Intervenção Coronária Percutânea
Source: Arq Bras Cardiol. 2026 Feb 26;123(1):e20260022. [Article in Portuguese] doi: 10.36660/abc.20260022 (PMC13128176; doi:10.36660/abc.20260022)
Supplement: Translation

# Translation: Reconsidering the CHA_2_DS_2_-VASc Score for No-Reflow Risk Stratification after Percutaneous Coronary Intervention

**DOI:** 10.36660/abc.20260022i

**Authors:** Irfan Sahin, Muhammed Furkan Deniz

**Affiliations:** 2 Istambul Turkey Bagcilar Training and Research Hospital, Istambul – Turkey

**Keywords:** Percutaneous Coronary Intervention, Postoperative Complications, No-Reflow Phenomenon

The no-reflow phenomenon remains one of the most complex and clinically challenging complications of percutaneous coronary intervention (PCI).^1 , 2^Although advanced imaging techniques, novel stent technologies, and contemporary pharmacological therapies are widely used today, there is still no universally accepted scoring system capable of reliably predicting no-reflow either before or during the procedure. This gap remains an important unmet clinical need.^1 - 4^

In this context, the systematic review and meta-analysis presented by Ozbay et al. make a significant contribution to the existing literature by addressing the association between the CHA_2_DS_2_-VASc score and the occurrence of no-reflow after PCI.^3^By synthesizing data from ten observational studies involving more than 6,500 patients, the authors demonstrated a significant and consistent association between higher CHA_2_DS_2_-VASc scores and an increased likelihood of no-reflow. These findings suggest that observations previously reported in single-center studies with relatively small sample sizes are confirmed in a larger population within a more robust methodological approach.^4 - 11^

The potential role of the CHA_2_DS_2_-VASc score in predicting no-reflow is supported not only by statistical associations but also by strong pathophysiological concordance. The score incorporates components such as advanced age, hypertension, diabetes mellitus, congestive heart failure, vascular disease, prior stroke, and female sex. All of these clinical characteristics are closely linked to endothelial dysfunction, chronic inflammation, increased thrombotic tendency, and microvascular disease.^4 , 12^Considering that distal embolization, microvascular obstruction, vasoconstriction, and reperfusion injury are key mechanisms involved in the pathogenesis of no-reflow, the CHA_2_DS_2_-VASc score may serve as a comprehensive clinical surrogate reflecting these underlying processes.^2^Therefore, the use of this score in the context of post-PCI no-reflow should be viewed not as an incidental extension but as a biologically meaningful repositioning.

One of the notable strengths of the meta-analysis is the overall consistency of results across different clinical subgroups. Despite a high degree of heterogeneity, the maintenance of effect direction in sensitivity analyses and multiple subgroup evaluations suggests that the observed association reflects a systematic pattern rather than a chance finding.^3^Subgroup analyses limited to patients with acute ST-segment elevation myocardial infarction (STEMI) also demonstrated a persistent and significant association between the CHA_2_DS_2_-VASc score and no-reflow.^5 - 7^Furthermore, the reduction in heterogeneity and maintenance of statistical significance after exclusion of studies reporting extreme effect sizes further support the robustness of the findings.

Although data on patients with non–ST-segment elevation myocardial infarction (NSTEMI) were more limited, the available evidence showed a trend consistent with the overall results of the meta-analysis. Studies demonstrating an association between the CHA_2_DS_2_-VASc score and no-reflow in NSTEMI populations require cautious interpretation because of smaller sample sizes and the observational nature of the data. These findings support the interpretation that the CHA_2_DS_2_-VASc score may serve as a risk marker across different clinical presentations within the acute coronary syndrome spectrum.

Nevertheless, careful interpretation of these findings is warranted. The observational nature of all included studies renders the analysis susceptible to residual and unmeasured confounding. Variables such as procedural techniques, operator experience, thrombus burden, lesion morphology, adjunctive pharmacological therapies, and strategies aimed at reducing microembolization were not uniformly reported across studies.^3^In addition, variations in the definition of no-reflow and differences in statistical modeling approaches represent important sources of heterogeneity.^3 , 11 , 13^The presence of publication bias and small-study effects also cannot be entirely excluded.

In light of these limitations, it is clear that the CHA_2_DS_2_-VASc score should not be regarded as an independent determinant or a decision-making tool for no-reflow. Rather, it should be considered a practical clinical indicator reflecting cumulative vascular risk burden and susceptibility to microvascular injury. In this context, the CHA_2_DS_2_-VASc score emerges as a tool suitable for risk enrichment rather than a diagnostic test.^3 , 11^

From a clinical perspective, in patients with higher CHA_2_DS_2_-VASc scores, the score may serve as an early warning signal prompting more meticulous procedural planning, implementation of strategies to minimize distal embolization, and closer post-procedural monitoring.^3 , 9^

Future studies should investigate whether integrating clinical scores such as CHA_2_DS_2_-VASc with angiographic findings, procedural variables, and inflammatory or thrombotic biomarkers can further improve the prediction of no-reflow.^3^More importantly, well-designed prospective randomized studies are needed to determine whether such integrated risk models translate not only into improved predictive accuracy but also into better clinical outcomes.

In conclusion, the meta-analysis by Ozbay et al. demonstrates a significant and consistent association between the CHA_2_DS_2_-VASc score and the development of no-reflow after PCI.^3^This finding further underscores the close relationship between systemic vascular risk and microvascular injury and suggests that the CHA_2_DS_2_-VASc score may help identify patients at increased risk of no-reflow following PCI. Given the multifactorial etiology of no-reflow and its association with serious clinical outcomes, the CHA_2_DS_2_-VASc score may represent a useful tool for identifying high-risk patients.
